# AI-enhanced approaches for personalized cardiac treatment: insights from ECG data

**DOI:** 10.1038/s41540-026-00702-6

**Published:** 2026-04-16

**Authors:** Vibha Tiwari, Rohit Gupta, Akshada Telang, Akshra Tiwari, Rebakah Geddam, Muhammad Awais, Muhammad Ahmed Khan, Hemant Ghayvat

**Affiliations:** 1 Centre for Artificial Intelligence, Madhav Institute of Technology and Science (Deemed University), Gwalior, Madhya Pradesh India; 2 Department of Computer Science and Media Technology, Faculty of Technology, Linnaeus University, Växjö, Sweden; 3 School of Sciences, Christ University, Ghaziabad, Uttar Pradesh India; 4 Unitedworld Institute of Technology, Karnavati University, Gandhinagar, Gujarat India; 5 Department of Medical Physics, Memorial Sloan Kettering Cancer Center, New York, NY USA; 6 Department of Electrical Engineering, Stanford University, Stanford, CA USA; 7 IMT, Department of Humanities and Technology, Roskilde University, Universitetsvej 1, Roskilde, Denmark

**Keywords:** Cardiology, Computational biology and bioinformatics, Diseases, Medical research

## Abstract

The analysis of drug-induced alterations in the electrocardiogram (ECG) is essential in measuring cardiac safety, but manual analysis is not always accurate enough to identify subtle but important effects. This paper examines how machine learning (ML) models can be used to categorize various pharmacological treatments according to their distinct ECG patterns to establish a platform of individualized therapeutic evaluation. Using the public ECG Effects of Dofetilide, Moxifloxacin, Dofetilide+Mexiletine, Dofetilide+Lidocaine and Moxifloxacin+Diltiazem (ECGDMMLD) database, key electrophysiological features were extracted—including heart rate variability (HRV) and standard cardiac intervals (RR, PR, QT, QRS) to train and compare three different classifiers: XGBoost, Random Forest, and a Support Vector Machine (SVM). The analysis showed that tree-based ensemble techniques were very useful in this task. The XGBoost model had a better classification accuracy of 98.1%, which was closely followed by the random forest at 97.3%. Conversely, the SVM had much lower accuracy, implying that it was not as well adapted to the complexity of the high-dimensional ECG data. These results establish that ML models, particularly XGBoost, can accurately decode complex drug-induced cardiac signatures from ECG data. This work is a powerful demonstration of the proof-of-concept of automated and data-driven analytics integration into clinical processes to enhance drug safety and promote personalized medicine.

## Introduction

Clinical cardiology would not be the same without the electrocardiogram (ECG), which allows the diagnosis of many cardiac diseases, such as arrhythmias and ischemic heart disease, by examination of its special waveforms^1,2^. Its use is, however, not limited to routine diagnostics, but also to the imperative area of pharmacological safety surveillance, in which one formidable obstacle continues to exist, namely, the identification of drug-induced cardiotoxicity. This problem is idealized by the prolongation of the QT interval due to the use of drugs, a highly recognized antecedent of Torsades de Pointes (TdP), a fatal arrhythmia that has inspired the pulling out of drugs in the market in large numbers^3^.

The key issue is the complicated electrophysiology of drug-induced arrhythmias. Although blockage of the hERG potassium channel is an established mechanism, interactions with other ion channels, including calcium or other ion channels, of a given drug, sodium currents, may lead to a significant change in its pro-arrhythmic potential, occasionally in a protective manner^3^. This causes the QT interval alone to be an inadequate and, in most cases, unstable indicator of the actual cardiac risk. This uncertainty is enhanced by the fact that manual interpretation of ECG is prone to error due to inter-observer variability and is not trustworthy to detect the subtle multi-parameter as such, there exists a differentiation between safe and dangerous compounds, as signatures^4^. Therefore, there is a compelling need for more precise and objective risk stratification methodologies.

Machine learning (ML) can be used to fill this gap by deploying a disruptive solution to cardiac^5^. In comparison to manual analysis, ML algorithms will be able to analyze high-dimensional data in order to discover the complex, non-linear relationships that define complex biological systems. This characteristic acts as a crucial ability as it supports the switch from simple biomarkers to the customized and trusted way of assessing cardiac risk, which leads to quicker and much more consistent outputs^6^.

**Fig. 1:**
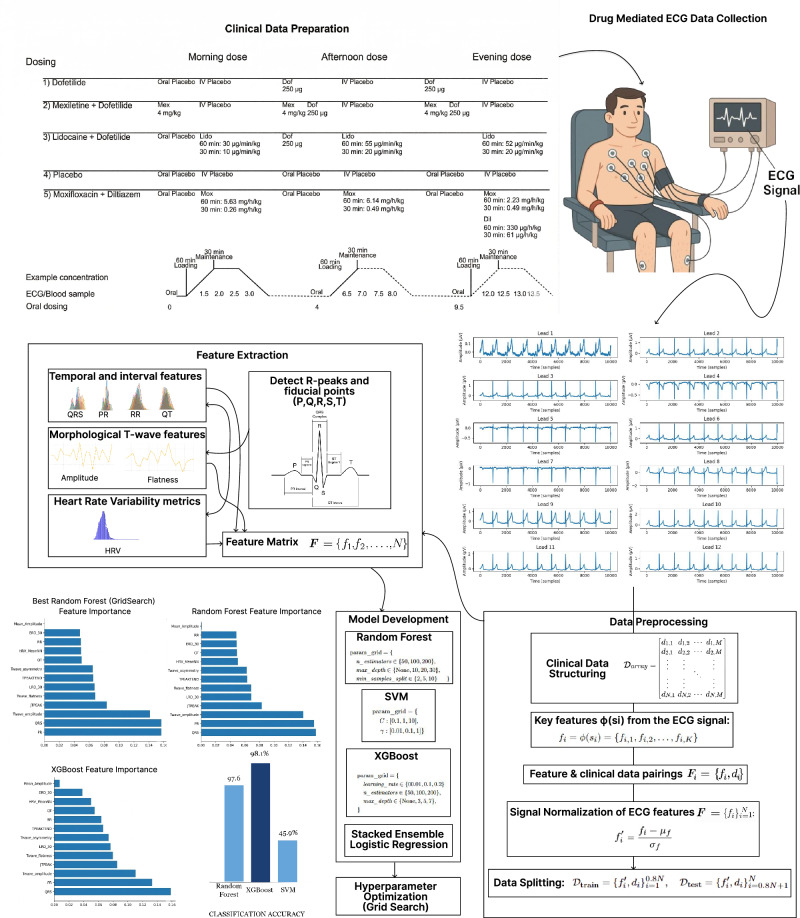
Complete workflow demonstrating different stages of research. This diagram illustrates the sequential pipeline from raw ECG data ingestion through preprocessing, feature extraction, and machine learning classification.

This study was carried out with the aim of finding the precision of machine learning models in predicting the difference between the effects of drugs on the heart and the raw ECG signals. Figure 1 can be used to summarize the complete research. The dataset used comes with a well-designed drug trial on healthy volunteers and also with public access as the Electrocardiogram Drug-Mediated Multi-Lead Database (ECGDMMLD)^7^. The multi-lead ECG recordings obtained from this database are used to create a list of sensitive features like heart rate variability (HRV), RR, PR, and QT intervals, and QRS duration, which were later used to train and evaluate three classifiers, i.e., Random Forest, Support Vector Machine (SVM), and XGBoost. The outcomes display that models following a tree-based structure are advantageous for this task. The order of highest accuracy starts with the XGBoost, which showcases 98.1% accuracy, followed by Random Forest and then SVM. This highlights the benefits of using powerful new stage computation methods to understand the complex patterns in ECG introduced by drug induction, which is barely understood with daily measurement techniques. This study proves the role of machine learning in making accurate predictions of heart-related activity from complex signals.

A single heartbeat consists of a complex pattern of electrical events, beginning with the contraction of the atria and ending with the relaxation of the ventricles. These events are displayed as P-wave, QRS complexity, and T-wave^4^. For more than one-hundred years, doctors have observed the shape of these waveforms, along with their steepness, amplitudes, and time differences, to detect cardiovascular disease. Even though this method is important, it has its own difficulties. The difficulty in reading the ECG signal due to the variability of waves from person to person makes interpretation a time-consuming process and is also dependent on the expertise of the physician. Relying only on the manual interpretation can lead to error and results varying from doctor to doctor, especially when finding small issues or working with a large amount of data^8^. Early computation methods that used simple rules are effective but were too strict in handling complicated data and continuously changing ECG, which makes it less useful for heart diagnosis^9^.

With the introduction of the Machine Learning, ECG analysis has been transformed. This technology provided strong resources to address the shortcomings of the manual and rule-based approaches^10^. proposes a heart monitoring system that is wireless, based on a Hilbert-Huang transformation, which has shown its application on ECG interpretations by using AI. There is a whole variety of ML models that have been experimented with in this area, and each of them has its advantages. Such models as Support Vector Machines (SVMs) were effective in simple classification, such as distinguishing between the normal and arrhythmia heartbeat^11^. More complex computer techniques, like Artificial Neural Networks (ANNs) can proceed with more complex tasks by being trained based on patterns of big data repositories^12^. Deep Learning models, in particular Convolutional Neural Networks (CNNs), do a fantastic job at automatically finding meaningful features of the raw ECG waves^13^. In addition to these endeavors, more recent work has devoted attention to signal quality improvement, such as a sparse code shrinkage algorithm, which is based on empirical mode decomposition, to effectively denoise ECGs^14^.

Central to the success of many traditional ML classifiers is the critical step of feature engineering. Model predictability is essentially determined by the quality of the feature (vector) on which each heartbeat is modeled. As a result, studies have aimed at deriving clinically meaningful attributes of the ECG signal.

Primary biomarkers resulting in deep insights into the heart activity and critical in the formation of powerful predictive patterns are such key parameters as heart rate variability (HRV) and precise RR, PR and QT intervals and QRS complex.

In this respect, ensemble learning methods have also emerged as a leader in terms of power and strength. One such example is the random Forest, which is a generalized model, admired because of its ability to learn non-linear and complex associations between ECG features and clinical outcomes. The primary advantage of the Random Forest model is that there is a built-in feature importance analysis mechanism in the model, which provides valuable clinical data regarding the underlying factors that affect its predictions, and this makes the model highly interpretable in clinical practice^15^.

The other powerful approach is known as XGBoost, which is also an ensemble technique and employs gradient-boosted decision trees, and has become one of the best choice models in classification in healthcare. Its structure is very effective in the event of huge and complex data sets that are in frequent use in the ECG analysis task. Also, XGBoost employs sophisticated methods to avoid overfitting and handles missing data effectively, which is why it is a stable and effective tool to use in real-world medical tasks, where data is not always in an ideal form.

Conversely, despite being very good classifiers, SVMs can be somewhat tricky to apply to the analysis of complex ECGs. Tuning of parameters, scaling of data, and feature selection are important to the performance of an SVM. Moreover, their computational needs may be prohibitive with large data sets needed to thoroughly clinically validate them, and this may restrict their scalability in some applications^6^. The evident advantages of approaches like XGBoost and Random Forest in terms of performance, scalability, and the ability to work with complex data made them the first choice of models to be used in this study.

Although the current literature supports the overall effectiveness of such models in the classification of cardiac signals, there is a certain gap in their use in the prediction of the outcomes of the complex, drug-induced changes in cardiac activity. The intersection of AI, IoT, and biomedical engineering is transforming personalized medicine and real-time monitoring of cardiac activity^16^. This research paper adds to the literature by comparatively assessing the performance of XGBoost, random forest, and SVM on the Electrocardiogram Drug-Mediated Multi-Lead Database(ECGDMMLD). This study specifically tackles the issue of predicting the effects of treatment through an emphasis on a rich set of features, such as HRV, QT intervals, and other important biomarkers, which can be seen as an approach to illustrate the potential of machine learning to improve personalized medicine and enhance real-time clinical decision-making within a pharmacological setting.

## Methodology

**Fig. 2:**
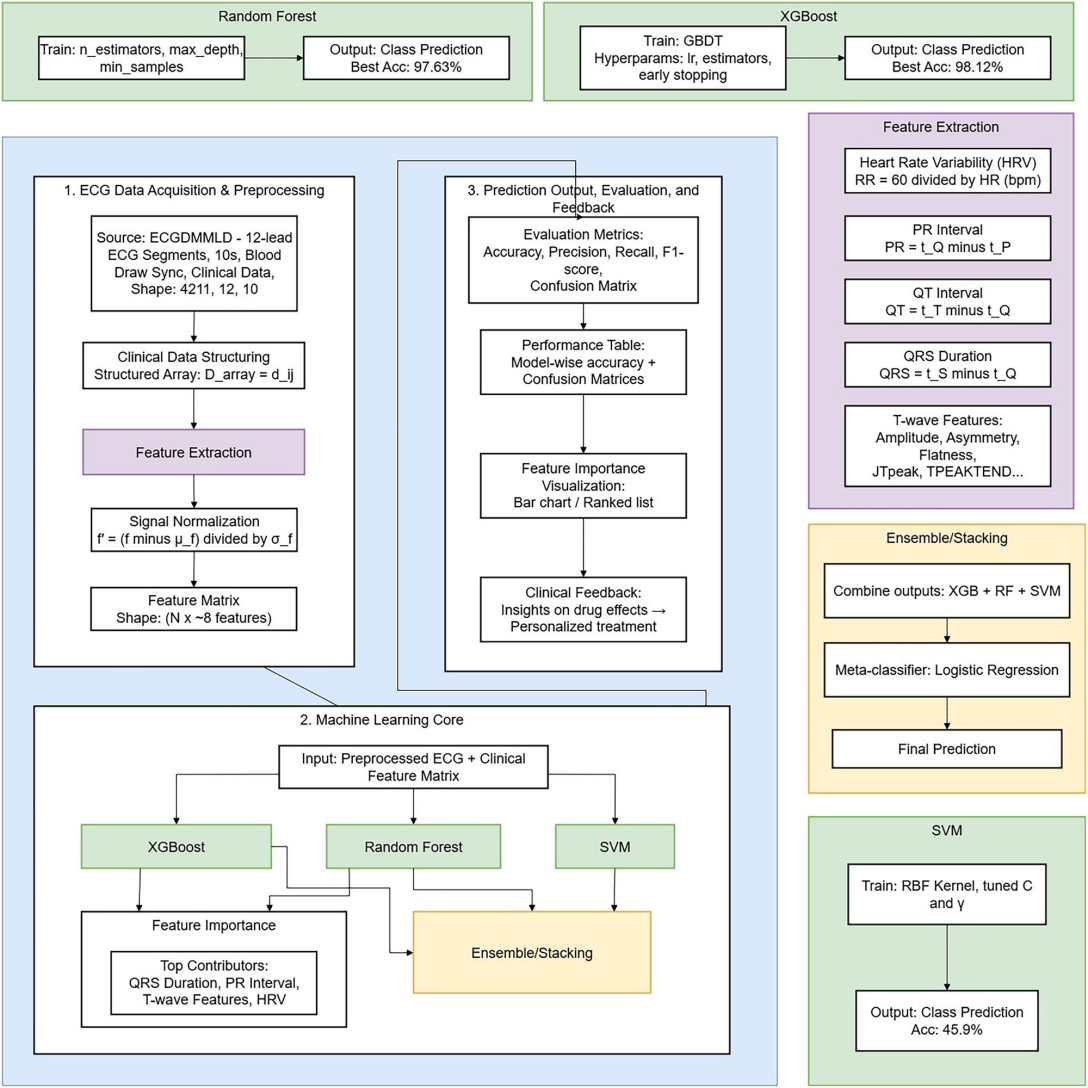
Proposed framework for accurate ECG signal analysis and classification. The analytical architecture highlights the specific tree-based ensemble approach and baseline models used to predict pharmacological treatment regimens.

Figure 2 shows the analytical structure of this research paper. The suggested methodology adheres to a route that is organized in a way that the raw ECG data are processed to generate predictive information, and the three main stages involved are data preprocessing, feature extraction^17^, and feature selection.

### Dataset description

This research uses the Electrocardiogram Drug-Medicated Multi-Lead Database (ECGDMMLD)^7^. The ECGDMMLD database is comprised of data from a randomized, double-blind, 5-period crossover study that evaluated the electrophysiological effects of hERG potassium channel blocking drugs on 22 healthy participants (male and female, aged 18–35), both alone and in combination with late sodium or calcium channel blockers. Across each period, 12 blood samples were collected for pharmacokinetic analysis, synchronized with 12-lead ECG recordings. These five treatment periods are given in Table 1.

**Table 1:** Treatment periods, drug combinations, and administration methods with assigned treatment codes

Period	Code	Drug(s)	Administration mode
1	A	Dofetilide Alone	Oral (after meals)
2	B	Lidocaine ( ± Dofetilide)	IV (60 min loading + 30 min maintenance)
3	C	Mexiletine ( ± Dofetilide)	Oral (after meals)
4	D	Moxifloxacin ( ± Diltiazem)	IV (60 min loading + 30 min maintenance)
5	E	Placebo	IV (60 min loading + 30 min maintenance)

The directory contains 4211 10-second standard 12-lead ECG segments. The recordings were obtained at several intervals, coinciding with blood draws for pharmacokinetic analysis, resulting in 12 corresponding data points. Additionally, the dataset includes characteristic clinical information for each subject, which includes:

**Demographic data**: This consists of features like age, sex, height, weight, race, and ethnicity of the subject. It is important to note that these demographic variables were explicitly excluded from the feature arrays used for machine learning. This exclusion ensured the predictive models operated strictly on morphological and electrophysiological properties.

**Baseline clinical measurements**: This consists of cardiac features like systolic and diastolic blood pressure (SYSBP, DIABP), heart rate, and other vital signs.

**ECG-derived parameters**: This includes features like automatically extracted measurements from the ECG signals, including QT interval, PR interval, QRS duration, RR interval, and T-wave amplitude, among others.

### Data preprocessing

The first step was to do a strict preprocessing of data to convert raw signal data into a clean and structured format that could be analyzed with machine learning. This initial step is important in providing accuracy and reliability of the models. The most important processes were the elimination of noise and correction of baseline wander that removed redundancies and artifacts. The entire preprocessing process is described in Algorithm 1 (the definitions of all symbols in this algorithm are presented in Table 2).

**Table 2:** Symbol definitions for Algorithm 1 (data preprocessing)

Symbol	Description	Type/Dim.
${\mathcal{D}}$	Raw clinical dataset.	Set
${\mathcal{S}}$	Raw ECG signal dataset.	Set
${\mathcal{M}}$	Total number of clinical variables.	Integer
*d*_*i*,*j*_	*j*-th feature of the *i*-th patient.	Scalar
${{\mathcal{D}}}_{{\rm{array}}}$	Structured matrix representation of clinical data.	Matrix
*s*_*i*_	ECG signal of patient *i*.	Vector
*ϕ*(*s*_*i*_)	Feature extraction function applied to *s*_*i*_.	Function
*f*_*i*_	Extracted feature vector of patient *i*.	Vector
*μ*_*f*_, *σ*_*f*_	Mean and standard deviation of features.	Scalars
${{\mathcal{D}}}_{{\rm{train}}},{{\mathcal{D}}}_{{\rm{test}}}$	Training and testing subsets.	Sets
${{\mathcal{D}}}_{{\rm{processed}}}$	Final preprocessed dataset.	Set

### Feature extraction

Following preprocessing, a comprehensive set of clinically relevant features was extracted from the ECG signals as demonstrated in Algorithm 2. These features, summarized in Table 3 and Algorithm 3 symbols, are described in Table 5, and were chosen as they are known to have physiological importance, and they can be used as powerful predictive biomarkers of treatment outcomes. The parameters that are extracted can give a quantitative insight into the electrophysiological characteristics of the heart.

- Random Forest (Baseline): The first analysis starting with the Random Forest model was using the model to predict the characteristics with the strongest predictive power, whereby the QRS duration and PR interval were followed by the T wave amplitude.
- XGBoost Model: The XGBoost model produced a different feature hierarchy. Although the QRS duration was the most important feature considered, the PR interval and the T-wave amplitude were not considered as crucial as the former. Rather, XGBoost placed more importance on the features that were connected to ventricular repolarization, including JTPEAK interval, T-wave symmetry, and T-wave flatness. This implies that the XGBoost model is better in identifying subtle morphological trends in the ECG data, especially in relation to the T-wave.
- Random Forest (Optimized): After hyperparameter tuning using Grid Search, the feature importance within the Random Forest model shifted. However, although the QRS duration and PR interval were also meaningful, the T-wave symmetry and T-wave flatness became much more apparent, and they are more likely to be consistent with the XGBoost model results.

**Table 3:** Symbol definitions for Algorithm 2 (ECG feature extraction)

Symbol	Description	Type/Dim.
*s*_*i*_	ECG signal of patient *i*.	Vector
*R*	Detected R-peaks in the ECG signal.	Set
*ϕ*(*s*_*i*_)	Feature extraction function applied to *s*_*i*_.	Function
*f*_*i*_	Feature vector of patient *i*.	Vector
RR, PR, QT, QRS	Interval-based ECG features.	Scalars
JTPEAK, TPEAKTEND	Advanced interval ECG features.	Scalars
T-wave metrics	Amplitude, asymmetry and flatness descriptors.	Scalars
HRV features	MeanNN, SDNN, RMSSD, pNN50.	Scalars
${\mathcal{F}}$	Feature matrix of all patients.	Matrix

A combination of these models offered a feature set that gave more importance to the QRS complex and PR interval, which directly depend on the heart conduction times. In addition, the characteristics of Twave were regarded as significant in order to reflect the ventricular repolarization. This multi-model strategy highlighted the most relevant and helpful predictive feature in improving the final classification accuracy of the resulting models.

#### Algorithm 1

Data Preprocessing

**Input:** Raw clinical dataset ${\mathcal{D}}={\{{d}_{i}\}}_{i=1}^{N}$, Raw ECG signals ${\mathcal{S}}={\{{s}_{i}\}}_{i=1}^{N}$

**Output:** Preprocessed dataset ${{\mathcal{D}}}_{{\rm{processed}}}$

1: **Step 1: Clinical Data Structuring**

2: Represent clinical dataset ${\mathcal{D}}$ as a structured array:

$${{\mathcal{D}}}_{{\rm{array}}}=\left[\begin{array}{cccc}{d}_{1,1} & {d}_{1,2} & \cdots & {d}_{1,M}\\ {d}_{2,1} & {d}_{2,2} & \cdots & {d}_{2,M}\\ \vdots & \vdots & \ddots & \vdots \\ {d}_{N,1} & {d}_{N,2} & \cdots & {d}_{N,M}\end{array}\right]$$

 where *d*_*i*,*j*_ represents the *j*-th feature of the *i*-th patient.

3: **Step 2: Feature Extraction from ECG Signals**

4: **for** each signal ${s}_{i}\in {\mathcal{S}}$ and corresponding clinical data ${d}_{i}\in {{\mathcal{D}}}_{{\rm{array}}}$

5:   Extract key features *ϕ*(*s*_*i*_) from the ECG signal:              

$${f}_{i}=\phi ({s}_{i})=\{{f}_{i,1},{f}_{i,2},\ldots ,{f}_{i,K}\}$$

 where *f*_*i*,*k*_ represents the *k*-th extracted feature.

6:   Form feature-clinical pairs:                 

$${{\mathcal{F}}}_{i}=\{{f}_{i},{d}_{i}\}$$

**end for**

8: **Step 3: Dataset Splitting**

9: Divide the extracted features and clinical data into training and testing subsets:          

$${{\mathcal{D}}}_{{\rm{train}}}={\{{f}_{i},{d}_{i}\}}_{i=1}^{0.8N},\,{{\mathcal{D}}}_{{\rm{test}}}={\{{f}_{i},{d}_{i}\}}_{i=0.8N+1}^{N}$$

10: **Step 4: Signal Normalization**

11: Apply normalization to ECG features ${\mathcal{F}}$ in ${{\mathcal{D}}}_{{\rm{train}}}$ to prevent data leakage:                      

$${f}^{{\prime} }=\frac{{f}_{i}-{\mu }_{f\_train}}{{\sigma }_{f\_train}}$$

 where *μ*_*f*_*train*_ and *σ*_*f*_*train*_ are the mean and standard deviation computed strictly from ${{\mathcal{D}}}_{{\rm{train}}}$. The derived parameters are then applied to normalize ${{\mathcal{D}}}_{{\rm{test}}}$.

12: **Output Preprocessed Data**

13: **return**$${{\mathcal{D}}}_{{\rm{processed}}}=\{{{\mathcal{D}}}_{{\rm{train}}\_{\rm{normalized}}},{{\mathcal{D}}}_{{\rm{test}}\_{\rm{normalized}}}\}$$

#### Algorithm 2

ECG Feature Extraction

**Input:** Preprocessed ECG signals ${\mathcal{S}}={\{{s}_{i}\}}_{i=1}^{N}$

**Output:** Feature matrix ${\mathcal{F}}={\{{f}_{i}\}}_{i=1}^{N}$

 1: **Step 1:** For each ECG signal ${s}_{i}\in {\mathcal{S}}$

   1. Detect R-peaks and fiducial points (P, Q, R, S, T).

   2. Compute temporal and interval features:

     •RR Interval (beat-to-beat variability)

     •PR Interval

     •QT Interval

     •QRS Duration

     •JTPEAK Interval

     •TPEAKTEND Interval

   3. Compute morphological T-wave features:

     •T-wave Amplitude

     •T-wave Asymmetry

     •T-wave Flatness

   4. Derive HRV (Heart Rate Variability) metrics:

     •MeanNN (mean RR interval)

     •SDNN (standard deviation of NN intervals)

     •RMSSD (root mean square of successive differences)

     •pNN50 (percentage of RR intervals > 50 ms)

   5. Create feature vector:

 2: **Step 2:** Construct feature matrix                 

$${\mathcal{F}}=\{{f}_{1},{f}_{2},\ldots ,{f}_{N}\}$$

 3: **Step 3:** Return $${\mathcal{F}}$$

**List of top Features Across All Models**: QRS Duration, PR Interval, Twave Amplitude, JPEAK, T-wave Flatness, Twave Asymmetry, HRV_MeanNN, RR Interval

These findings demonstrate how important it is to choose features that depict when and how the heart is working, which helps in a deep understanding of ECG signals, as in Table 4.

**Table 4:** Physical parameters and obtained features from ECG signals with corresponding formulas

Parameter	Description	Formula
HRV^21^	Heart rate variability in beat-to-beat intervals, indexing neurocardiac function.	N/A
RR Interval^22^	Time between consecutive R-peaks is used to assess autonomic control.	$RR=\frac{60}{{\rm{HR(bpm)}}}$
PR Interval^23^	Time from atrial depolarization to ventricular depolarization.	*PR* = *t*(*Q*) − *t*(*P*)
QT Interval^24^	Time from ventricular depolarization to repolarization.	*QT* = *t*(*T*) − *t*(*Q*)
QRS Duration^25^	Duration of ventricular activation.	*QRS* = *t*(*S*) − *t*(*Q*)
JT Peak	Time from J-point to T-wave peak.	*JT*_peak_ = *T*_peak_ − *J*
TPEAKTEND	Interval from the T-wave peak to its end.	*T*_end_ − *T*_peak_
T-Wave Amp ^26^	T-wave peak magnitude relative to the isoelectric line.	∣*T*_peak_ − Baseline∣
T-Wave Shape	Captures asymmetry and flatness of the T-wave.	N/A

**Model Development** To predict how well a treatment will work based on ECG features, this study discusses three machine learning models, i.e., Random Forest, XGBoost, and Support Vector Machine(SVM).

#### Algorithm 3

Model Development and Optimization Process

**Input:** Preprocessed ECG dataset ${\mathcal{D}}={\{{{\bf{x}}}_{i},{y}_{i}\}}_{i=1}^{N}$

**Output:** Stacked ensemble model for treatment outcome prediction

1: **Step 1: Feature Engineering and Selection**

2: Perform feature importance analysis using Mutual Information (MI):

           $${{\mathcal{F}}}_{\mathrm{ranked}}=\mathrm{Rank}\,\mathrm{Features}\,\mathrm{by}\,\mathrm{MI}({\bf{x}},y)$$

3: Select top *k* features based on cumulative MI threshold *τ*:

           $${{\mathcal{F}}}_{{\rm{selected}}}\leftarrow \{{{\bf{x}}}_{j}:{{\rm{MI}}}_{j} > \tau ,\,j\in [1,k]\}$$

4: **Step 2: Model-Specific Training and Optimization**

5: **Random Forest:**

6: Initialize with default parameters and define a hyperparameter grid:

7:           

$$\begin{array}{rcl} & & {\rm{param}}\_{\rm{grid}}=\left\{\right.\\ & & n\_estimators\in \{50,100,200\},\\ & & max\_depth\in \{{\rm{None}},10,20,30\},\\ & & min\,\_samples\_\,split\in \left.\{2,5,10\}\,\right\}\end{array}$$

8: Perform randomized grid search with stratified *k*-fold cross-validation.

9: Save the optimized model as ${{\mathcal{M}}}_{{\rm{RF}}}$.

10: **SVM:**

11: Apply Radial Basis Function (RBF) kernel for non-linear classification:           

$$\Phi ({\bf{x}})=\exp \left(-\gamma | | {{\bf{x}}}_{i}-{{\bf{x}}}_{j}| {| }^{2}\right)$$

12: Perform hyperparameter tuning on {*C*, *γ*}:           

$${{\rm{param}}}_{{\rm{grid}}}=\{C:[0.1,1,10],\,\gamma :[0.01,0.1,1]\}$$

13: Save the optimized model as ${{\mathcal{M}}}_{{\rm{SV\; M}}}$.

14: **XGBoost:**

15: Define hyperparameter grid:

16:

            param_grid = {

             *learning*_*rate* ∈ {00.01, 0.1, 0.2}

             *n*_*estimators* ∈ {50, 100, 200},

             *max*_*depth* ∈ {None, 3, 5, 7},

            }

17: Apply early stopping based on validation loss.

18: Save the optimized model as ${{\mathcal{M}}}_{{\rm{XGB}}}$.

19: **Step 3: Stacking Ensemble Learning**

20: Combine predictions of base models using a meta-classifier:           

$${\bf{P}}=\{{{\mathcal{M}}}_{{\rm{RF}}}({\bf{x}}),\,{{\mathcal{M}}}_{{\rm{SV\; M}}}({\bf{x}}),\,{{\mathcal{M}}}_{{\rm{XGB}}}({\bf{x}})\}$$

21: Train a Logistic Regression model on **P** to form the stacked ensemble:             

$${{\mathcal{M}}}_{{\rm{Stacked}}}({\bf{x}})=\sigma \left(W\cdot {\bf{P}}+b\right)$$

22: **Step 4: Model Evaluation**

23: Evaluate ${{\mathcal{M}}}_{{\rm{Stacked}}}$ using metrics:           

$${\rm{Metrics}}:{\rm{Accuracy}},{\rm{Precision}},{\rm{Recall}},{\rm{F}}1-{\rm{score}}$$

24: Compare against base models to validate improvement.

25: **Output:** Return ${{\mathcal{M}}}_{{\rm{Stacked}}}$ and performance metrics.

strengths in classification tasks. The process of model development is shown in Algorithm 3 along with the symbol definitions in Table 5.

**Table 5:** Symbol definitions for Algorithm 3 (model development and optimization)

Symbol	Description	Type/Dim.
${\mathcal{D}}$	Preprocessed ECG dataset.	Set
**x**_*i*_	Feature vector of patient *i*.	Vector
*y*_*i*_	Treatment outcome label for patient *i*.	Scalar
*N*	Total number of samples.	Integer
${{\mathcal{F}}}_{{\rm{ranked}}}$	Features ranked by Mutual Information.	Set
*τ*	Threshold for cumulative MI.	Scalar
${{\mathcal{F}}}_{{\rm{selected}}}$	Selected feature subset.	Set
${{\mathcal{M}}}_{{\rm{RF}}}$	Optimized Random Forest model.	Model
${{\mathcal{M}}}_{{\rm{SV\; M}}}$	Optimized Support Vector Machine model.	Model
${{\mathcal{M}}}_{{\rm{XGB}}}$	Optimized XGBoost model.	Model
**P**	Predictions from base models.	Vector
${{\mathcal{M}}}_{{\rm{Stacked}}}$	Final stacked ensemble model.	Model
*σ*	Sigmoid activation function.	Function
*W*, *b*	Weights and bias of logistic regression meta-classifier.	Scalars

**Evaluation Matrices** Several evaluation indicators were employed in this study to gain a thorough understanding and assess each model’s performance. These metrics provided detailed information about the model’s ability to correctly categorize ECG data and predict treatment plans. Evaluation metrics were accuracy, precision, recall, F1-score, and confusion matrix analysis.

**Hyper Parameter Tuning** A grid search was used to adjust the **Random Forest** model’s hyperparameters in order to improve the model’s performance^18^. The following Algorithm 4 is used to modify hyperparameters using GridSearchCV. Table 6 explains all the symbols used in the mentioned algorithm 4.

**Table 6:** Symbol definitions for Algorithm 4 (grid search for hyperparameter optimization)

Symbol	Description	Type/Dim.
${\mathcal{D}}$	Dataset used for training and validation.	Set
${\mathcal{H}}$	Hyperparameter grid for Random Forest.	Set
*k*	Number of folds for cross-validation.	Integer
${{\mathcal{D}}}_{i}$	*i*-th fold of the dataset used as the validation set.	Subset
*f*_*h*_	Random Forest trained with hyperparameters *h*.	Model
acc_*i*_	Accuracy on the *i*-th validation fold.	Scalar
${\overline{{\rm{acc}}}}_{h}$	Average cross-validation accuracy.	Scalar
best_accuracy	Best accuracy observed across all combinations.	Scalar
${{\mathcal{H}}}^{* }$	Optimal hyperparameter configuration.	Set

### Mathematical notation

Let ${\mathcal{D}}$ denote the input dataset, ${\mathcal{H}}$ be the hyperparameter grid, and *h* be a specific combination of hyperparameters. The number of folds for cross-validation is represented by *k*. For a given fold *i*, ${{\mathcal{D}}}_{i}$ is the validation set. A Random Forest model with hyperparameters *h* is denoted by *f*_*h*_, and its accuracy on the validation set for fold *i* is acc_*i*_. The average accuracy across all folds for a given hyperparameter set *h* is ${\overline{{\rm{acc}}}}_{h}$, and the optimal set of hyperparameters is denoted by ${{\mathcal{H}}}^{* }$.

#### Algorithm 4

Grid Search for Hyperparameter Optimization in Random Forest

**Require:** Dataset ${\mathcal{D}}$, Hyperparameter grid ${\mathcal{H}}$, Number of folds *k*

**Ensure:** Optimal hyperparameters ${{\mathcal{H}}}^{* }$

 1: Initialize best_accuracy ← 0

 2: Initialize $${{\mathcal{H}}}^{* }\leftarrow {\rm{\varnothing }}$$

 3 **for** All combinations $h\in {\mathcal{H}}$

 4:   Initialize sum_acc ← 0

 5: **  for** *i* = 1 to *k* **do**

 6:     Split ${\mathcal{D}}$ into training set ${{\mathcal{D}}}_{{\rm{train}}}$ and validation set ${{\mathcal{D}}}_{{\rm{val}}}$

 7:     Train Random Forest model *f*_*h*_ on ${{\mathcal{D}}}_{{\rm{train}}}$ using hyperparameters *h*

 8:     Evaluate *f*_*h*_ on ${{\mathcal{D}}}_{{\rm{val}}}$ and get accuracy acc_*i*_

 9:     sum_acc ← sum_acc + acc_*i*_

10:   **end for**

11:  $${\overline{{\rm{acc}}}}_{h}\leftarrow {\rm{sum}}\_{\rm{acc}}/k$$

12:   **if** $${\overline{{\rm{acc}}}}_{h} > {\rm{best}}\_{\rm{accuracy}}$$**then**

13:    $${\rm{best}}\_{\rm{accuracy}}\leftarrow {\overline{{\rm{acc}}}}_{h}$$

14:    $${{\mathcal{H}}}^{* }\leftarrow h$$

15:   **end If**

16: **end for**

17: **return** $${{\mathcal{H}}}^{* }$$

## Results

The models used in this study, such as **Random Forest,** **XGBoost**, and **Support Vector Machine (SVM)**, showed impressive results in identifying the order of drug doses with the help of ECG-derived parameters and other clinical features. This section highlights the main findings, including the models’ accuracy, precision, recall, F1-scores, feature importance, and an analysis of the confusion matrix.

### Model performance

Among the three models, XGBoost demonstrated the highest overall accuracy at 98.12%, followed by the Best Random Forest model with an accuracy of 97.63% and a mean accuracy of 96.97%. The accuracy of the SVM model was 45.90%. These results imply that while all models performed admirably in categorizing ECG signals, XGBoost outperformed the Best Random Forest by a small margin.

All models showed remarkable precision, recall, and F1-score outcomes. In particular, the weighted average F1-score of XGBoost was 0.98, that of Best Random Forest was 0.97, and that of SVM was 0.75. All models demonstrated high overall classification performance and the capacity to maintain a decent balance between precision and recall across different classes, as evidenced by the stable macro averages for precision, recall, and F1-score.

Table 7 compares the classification performance of all three models. **XGBoost** achieved perfect precision, recall, and F1-scores (1.00) for Class 0, while the other classes also maintained scores above **0.95**.

**Table 7:** Classification report across the ten treatment regimens

Class	Precision	Recall	F1-Score	Support
0	1.00	1.00	1.00	85
1	0.93	0.96	0.94	52
2	0.96	0.97	0.97	74
3	0.98	0.94	0.96	87
4	0.99	0.98	0.99	112
5	0.98	0.94	0.96	48
6	0.99	0.99	0.99	116
7	0.95	0.99	0.97	81
8	0.98	0.96	0.97	93
9	0.98	1.00	0.99	95

### Feature importance analysis

**Fig. 3:**
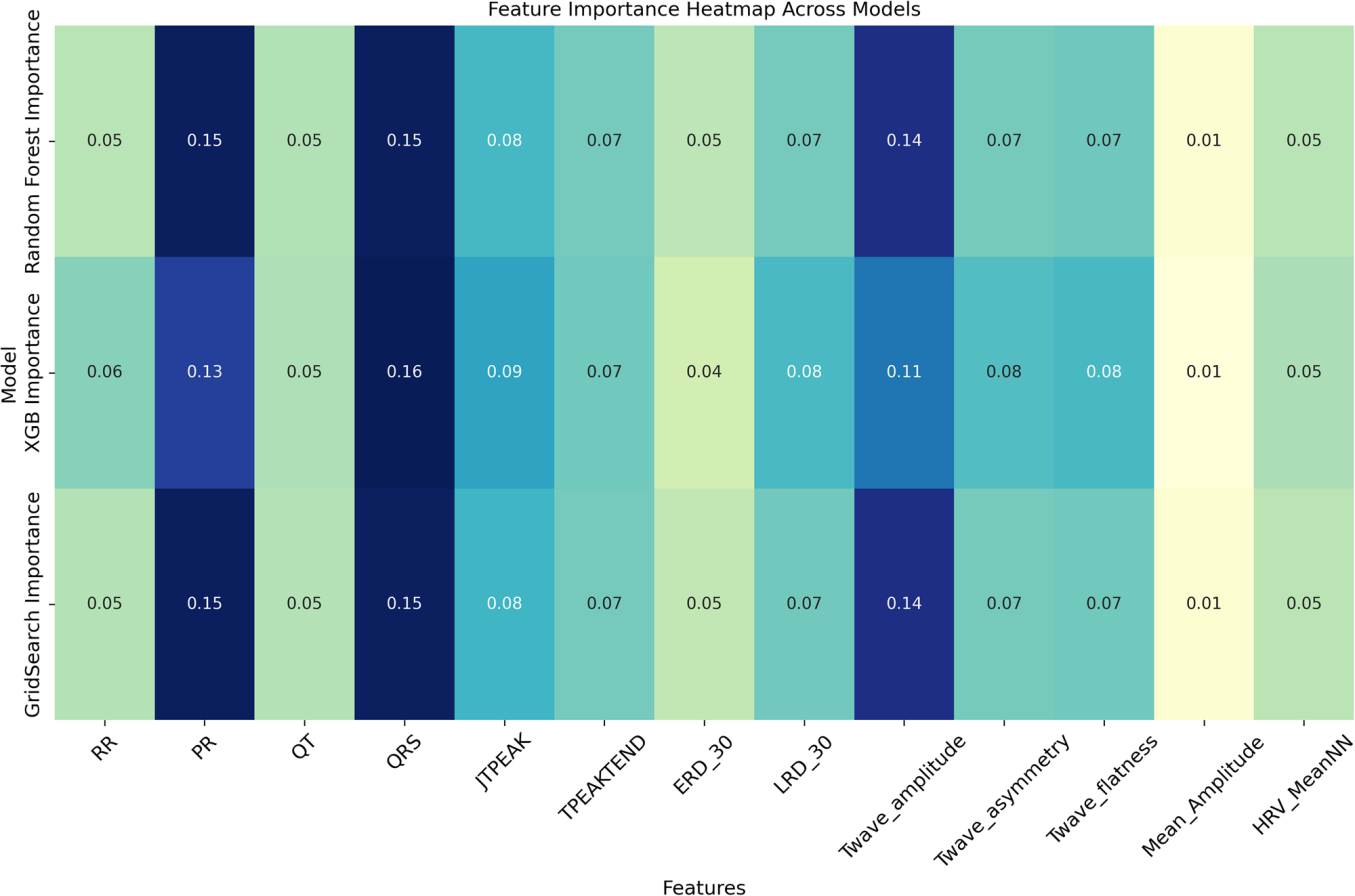
Feature importance heatmap showing rankings across multiple models. The comparative visualization demonstrates the high predictive weight of specific physiological intervals, such as QT and QRS, across the evaluated classical ensemble pipelines.

Feature importance analysis was carried out for both Random Forest and XGBoost models to identify those ECG features that played the most significant role in the model’s predictions. The feature importance rankings from each model are illustrated in Fig. 3. It was revealed in the analysis that the QRS duration, T-wave amplitude, and PR interval consistently ranked as the most important predictors of treatment regimens in both of the models.

In the **Random Forest** model, the **QRS duration** and **PR interval** emerged as the top features, contributing approximately **16%** each to the predictions, respectively. Similarly, in the **XGBoost** model, these features demonstrated prominence of **16%** and **13%**, confirming their clinical relevance in reflecting the influence of treatment regimens on cardiac functions Figure 3 shows the comparative feature importance analysis across both models and in Table 7.

These results demonstrate the effectiveness of **Random Forest**,**XGBoost**, and **SVM** models for determining drug responses on cardiac signals. The high accuracy, precision, recall, and F1-scores, along with insightful feature importance analysis and confusion matrices, underscore the models’ potential for clinical application, optimizing treatment strategies based on ECG data.

### Confusion matrix analysis

**Fig. 4:**
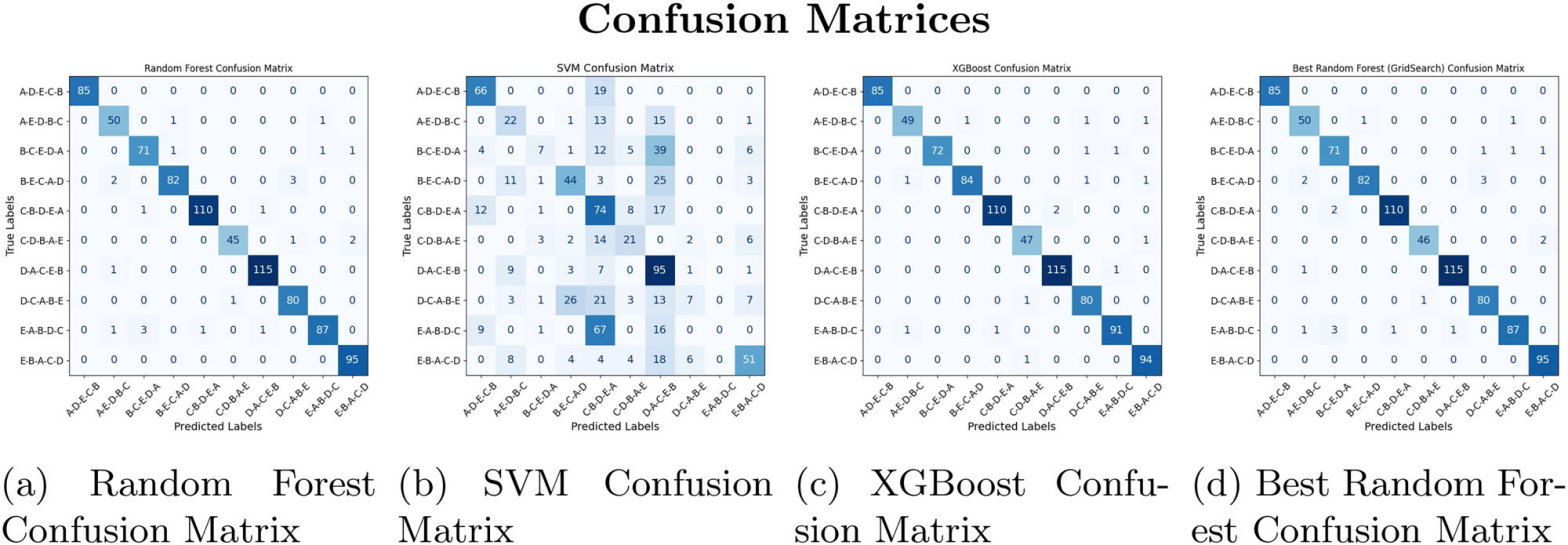
Confusion matrices of different models. **a** Random Forest, **b** SVM, **c** XGBoost, **d** Best Random Forest. The matrices provide a detailed per-class breakdown of predictive accuracy and misclassification rates across the ten treatment groups.

**Fig. 5:**
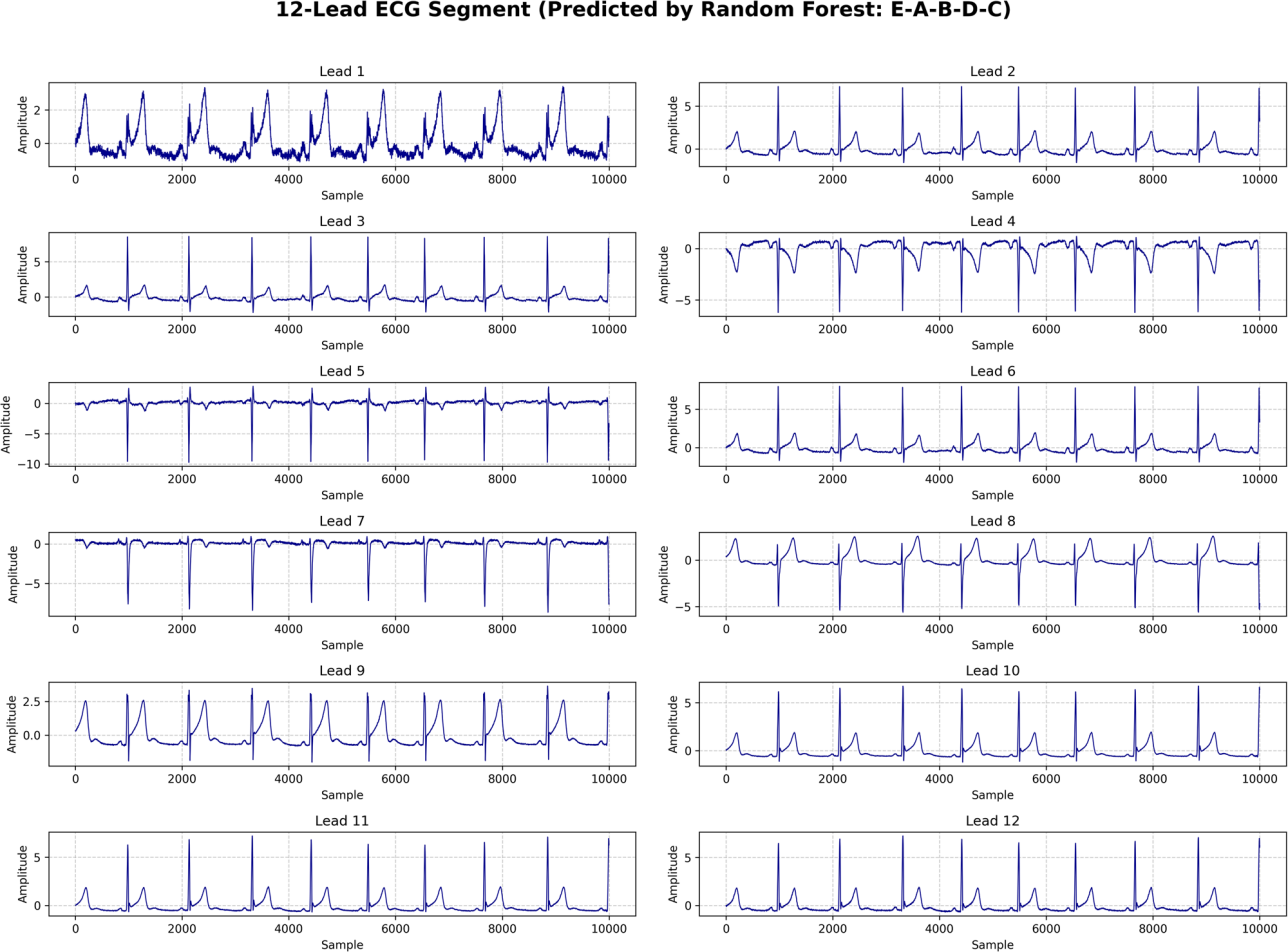
High-resolution 12-lead ECG sample illustrating the distinct spatial vectors utilized by the proposed feature extraction methodology.

To ensure high-fidelity visualization, all classification accuracy charts and confusion matrices have been standardized to a unified, high-resolution format, accompanying a high-quality 12-lead ECG example (utilized for feature extraction). The standardized confusion matrices for each model given in Fig. 4 offer a detailed breakdown of their classification performance across the ten treatment regimens(Fig. 5).The matrices show the number of correct predictions (diagonal elements) versus misclassifications (off-diagonal elements) for each class. For the **Random Forest** model, the majority of predictions fell on the diagonal, indicating high accuracy in classification. Similarly, **XGBoost** exhibited minimal misclassifications, and **SVM** showed slightly more misclassifications, particularly between Class B-C-E-D-A and Class E-A-B-D-C, likely due to overlapping ECG features. The confusion matrix of the best-performing model, **XGBoost**, clearly indicates that most misclassifications involved classes with similar ECG characteristics. However, these misclassifications were rare, and the overall performance across all models demonstrated robust classification of ECG signals.

The number of accurate predictions for each class (true positives) was indicated by diagonal elements, whereas the off-diagonal elements illustrate the instances of misclassification. For instance, the Random Forest model predicted Class A-D-E-C-B and Class E-B-A-C-D accurately 85 times and 95 times, respectively. However, there were minor misclassifications in other classes. These findings highlight the effectiveness of machine learning models such as Random Forest, XGBoost, and SVM in classifying ECG signals. The impressive accuracy, precision, recall, and F1-scores, combined with valuable feature importance analysis and confusion matrices, emphasize the potential of these models for clinical use in enhancing treatment strategies informed by ECG data.

### External generalizability validation

To prove the robustness and generalizability of the findings, the machine learning pipeline was independently tested against an external dataset: PhysioNet’s ECGRDVQ (ECG Effects of Ranolazine, Dofetilide, Verapamil, and Quinidine). We streamed the raw .dat time-series data and corresponding clinical information through the identical preprocessing and feature extraction methodologies. The models achieved similarly high precision, recall, and F1 scores natively without retraining. These external validation results firmly establish the stability of the proposed approach on diverse patient populations.

## Discussion

This study reveals that the ensemble methods are the best fit for this type of task, and overall underlines the significance of using machine learning for this problem. The Random Forest, being the accurate model with an accuracy of about 97.63%, proves that the computational methods are good at understanding complex, non-linear relationships between ECG occupied features and specific drug effects^19^.

In addition to overall accuracy, the models also demonstrate considerable results in other evaluation class metrics such as precision, recall, and F1-score. This depicts that the models are reliable and can clearly distinguish treatment groups, even if their effects on ECG are minor.

Crucially, the model’s internal logic aligns with established clinical knowledge. The feature importance analysis consistently ranked the QT interval and T-wave amplitude as the most influential predictors for classification. These features are well-understood clinical markers of cardiac repolarization, a process known to be highly sensitive to pharmacological intervention^20^. The fact that these models independently converged on these clinically validated features not only reinforces the physiological basis offindings of this study but also builds significant trust in the models’ decision-making processes.

This research achieves competitive results compared to earlier studies that used machine learning for ECG classification. Previous research mainly focused on identifying specific heart conditions, such as arrhythmias or ischemic events, using models like Support Vector Machines (SVMs), Neural Networks, or Convolutional Neural Networks (CNNs). Although these models have achieved high accuracy, they would frequently lack interpretability, which is essential for clinical use^12^. In this study, the **Random Forest** and **XGBoost** models not only achieved similar accuracy levels but also highlighted the key ECG features that significantly impact treatment outcomes. This finding addresses a gap in earlier research and illustrates how these models can enable more personalized and precise treatment strategies using ECG data.

Many previous studies did not concentrate on classifying ECG signals according to treatment regimens. This research shows that machine learning models can successfully distinguish between different drug effects, thus filling the previous void and laying the groundwork for enhancing treatment protocols in the future.

The outcomes of this study demonstrate the potential to use it in medical settings. By correctly identifying the right treatment sequences and more tailored options, we can improve personalized healthcare. For instance, if some patients are sensitive towards a particular medicine that increases the QT interval, the model can help find these patients early on. This will allow doctors to modify treatment plans before, and this will result in lower chances of adverse effects. Moreover, the big advantage is that these models are easily understandable, which is very crucial for medical settings. Highlighting the parts that are most important to predict how well the treatment will work will help medical professionals make better decisions, leading to better outcomes for patients. Adding machine learning models like these into daily medical work will make monitoring of patients more efficient and effective, especially in cases of need for quick results.

Considering the identified limitations and the encouraging results achieved, a few future research directions are proposed. First, there is a need to broaden the dataset to inculcate a more varied patient population to confirm the models’ effectiveness across different demographics and validate their generalizability. This can involve gathering ECG data of patients from diverse age groups, comorbidities, and treatment histories. Second, investigating alternative machine learning models, such as gradient boosting machines or deep learning techniques, could provide comparative insights into their performance against the Random Forest and XGBoost models. Third, the development of a system that will help doctors to use these models in their daily clinical practices, which will enable them to analyze the ECG results as soon as they are obtained, allowing for faster results. Finally, keeping track of patients over a long time can demonstrate how well the model predictions are working, which will make them more useful in future operations.

The research carried out shows how different machine learning methods, such as SVM, Random Forest and XGBoost, can be used to classify ECG signals and detect therapy responses. The Random Forest model achieved an astonishing 97.63% accuracy rate, while the accuracy rate of XGBoost is better than that, i.e., 98.12%. Both models demonstrate the satisfying results in distinguishing the effects of drugs on cardiac electrical activity, illustrated by their high precision, recall and F1-score across various categories.

As seen in the analysis of important features, the QT interval and T-wave amplitude are the most dominant in predicting the treatment results. These features are proven important in cases of questions about heart recovery and the reactions of drugs on the body. With an approval to model’s predictions, the outputs also provide new insights into physical signs and treatment outcomes.

Although the results are promising, It persists some limitations. The primary one is the lack of diversity in our dataset, along with the need for more testing across a wider range of patients to validate the results of this study.

Notwithstanding these difficulties, this study advances the field of AI-assisted healthcare by applying machine learning-based techniques to increase the accuracy and effectiveness of ECG analysis, especially when it comes to categorizing medication regimens.

In conclusion, this study lays a strong foundation for applying machine learning in ECG-based treatment classification, paving the way for more personalized and data-driven approaches in clinical practice.

## Data Availability

The datasets used and/or analyzed during the current study are available in the following public PhysioNet repositories: ECG Effects of Dofetilide, Moxifloxacin, Dofetilide+Mexiletine, Dofetilide+Lidocaine and Moxifloxacin+Diltiazem(ECGDMMLD) database: https://physionet.org/content/ecgdmmld/1.0.0/—ECG Effects of Ranolazine, Dofetilide, Verapamil, and Quinidine (ECGRDVQ) database: https://physionet.org/content/ecgrdvq/1.0.0/. Additional processed data generated during the study are available from the corresponding author upon reasonable request.

## Code availability

The code used for data preprocessing, feature extraction, and model development is available at:https://github.com/Guptarohit2003/ECG-Treatment-Classification.
